# Schoolchildren’s autobiographical memory: *COMT* gene Val^158^Met polymorphism effects on emotional content and quality of first memories

**DOI:** 10.1007/s10339-021-01064-z

**Published:** 2021-11-09

**Authors:** Pirko Tõugu, Tiia Tulviste, Toomas Veidebaum, Jaanus Harro

**Affiliations:** 1 Institute of Psychology, University of Tartu, Näituse 2, 50409 Tartu, Estonia; 2 National Institute for Health Development, Tallinn, Estonia; 3 Chair of Neuropsychopharmacology, Institute of Chemistry, University of Tartu, Tartu, Estonia

**Keywords:** *COMT* gene Val^158^Met polymorphism, First memories, Autobiographical memory, Schoolchildren, Gender differences

## Abstract

Autobiographical memory is a cognitive function strongly related to emotional processing as autobiographical memory often includes emotional content. The *COMT* gene Val^158^Met polymorphism is associated with both cognitive and emotional processing. *COMT* gene Val^158^Met polymorphism effects on the emotional content and quality of Estonian schoolchildren’s first autobiographical memories were investigated in the present study. In addition, gender effects were considered and the emotional valence of the first memory was taken into account. Schoolchildren’s (*N* = 234) first memories were coded for valence, emotion words, specificity, and details. Girls were more likely to provide specific memories and recollections with an emotional valence than boys were. Children described memories with a positive or a negative valence in more detail than neutral memories. Interactions between the COMT gene Val^158^Met polymorphism and gender and valence of the events were detected: Val/Met heterozygotes provided fewer details for emotional events; Val/Met heterozygote boys reported fewer details for their first memories than Val/Met heterozygote girls did; Met/Met homozygote children provided fewer evaluative details for emotional events.

## Introduction

Autobiographical memory is autonoetic episodic memory constituting a part of the declarative memory (Tulving 2002). It is also a basic human cognitive capacity strongly related to emotional processing, as autobiographical memory often includes emotional content. The *COMT* gene Val^158^Met polymorphism has been related to both cognitive and emotional processing; therefore, its role in autobiographical memory formation could be significant. The present study aims at establishing how the *COMT* gene Val^158^Met polymorphism is related to the details and emotional content of the first autobiographical memories remembered by Estonian schoolchildren.

### First autobiographical memories

Autobiographical memory skills develop alongside other cognitive attainments and are influenced by socialization (e.g., reminiscing with parents) throughout the preschool years (Nelson and Fivush 2004). The cognitive processes for remembering and recalling also become more efficient and effective during childhood (Bauer 2015). Autobiographical memory is most often defined as memory for specific one-time events (Fivush et al. 2011; Tulving 2002), and most people date the first events they can recall to an age of three to four years (see Bauer 2015, for review). The phenomenon of childhood amnesia has led researchers to propose several qualities of early memories that may enhance their survival: Early memories are often described as specific (Mullen 1994) and emotional (Howes et al. 1993).

The majority of people usually report specific events as their first autobiographical memories (Mullen 1994). Specific memories are contrasted to general memories and include at least one particular event that does not last longer than a day. General memories could include descriptions of routine events, or prolonged periods. At the same time, not all first recollections are of one-time specific events. Nuttall et al. (2014) have shown that children’s ability to provide specific one-time events increases with age and on the average only about half of the memories that 4–6-year-olds recall are specific. Studies suggest that memories, especially early memories, tend to become more generalized over the years. The kindergarten memories of school-age children reflect more general experiences than school memories (Picard et al. 2009), and general qualities are prominent in young adult’s very early first recollections (Howes et al. 1993). Women report more specific memories in their life histories compared to men (Pillemer et al. 2003).

Several studies have suggested that the first autobiographical memories are mostly of emotional events (Howes et al. 1993; Kihlstrom and Harackiewicz 1982). At the same time, other studies suggest that affect or emotion is often not explicitly mentioned in recollections (Mullen 1994). Several studies have identified gender differences in reporting emotions in first memories. Women’s recollections include more emotions they experienced during the event (Bauer et al. 2003; Fuentes and Desrocher 2013; Mullen 1994) and use more affective language describing past events (Rice and Pasupathi 2010) as compared to men. All children recall details about their own actions, other people, and artifacts involved in the events (Tõugu et al. 2014). At the same time, girls’ memories include more inner state talk (emotions and cognitions) than those of boys during the preschool years (Fivush et al. 1995) and later (Buckner and Fivush 1998).

Autobiographical memories include episodic and semantic components (Levine et al. 2002). The episodic details refer to the particular one-time-event at a specific place, while semantic details comprise knowledge about the world and oneself in it (Levine et al. 2002). Young children aged 5–6 years usually provide only the core episodic information; 8–9-year-olds report a fuller episodic account of the event and add evaluative information, i.e., they more often include a personal perspective of the event by including a modifier, intensifier (“*It was very, very long*.”), or by commenting on internal states (“*I liked it*.”) (Bauer and Larkina 2014). Women have been shown to recollect more details regarding the personal events (MacDonald et al. 2000; Wang 2013; Wang et al. 2011), particularly specific episodic details as compared to general semantic details (Wang et al. 2011). Conflicting findings regarding gender differences have been reported in studies aimed at children’s autobiographical memories. Some studies have shown that the preschool-age girls’ (Fivush et al. 1995; Pasupathi and Wainryb 2010) and women’s (Bauer et al. 2007; Buckner and Fivush 1998) personal narratives are reported in more detail than those of boys and men. Other studies have shown that the recollections of preschool boys and girls do not differ in detail (Wang 2004, 2008) or in episodic information (Tustin and Hayne 2010).

Childhood amnesia is already observable during middle childhood, as during that period children forget earlier events at an exponential rate (Bauer 2015). Children at the age of 8 and 9 begin to display an adult-like distribution of childhood memories (Bauer and Larkina 2014). Bauer suggests that many of the memories that children provide as their first in middle childhood have a potential of being retained as the first memory in adulthood (Bauer 2015; Bauer and Larkina 2014). Hence, middle childhood is a potent period to study the formation of adult-like autobiographical memory. Including genetic markers in the study may also help to shed light on some of the individual differences in autobiographical memory research.

### The ***COMT*** gene Val^158^Met functional polymorphism

Catechol-O-methyltransferase (COMT) is an enzyme that participates in the catabolism of catecholamines, including dopamine, and is associated with the functioning of the prefrontal cortex (Tunbridge et al. 2004). The COMT gene contains a functional polymorphism in codon 158, where methionine (Met) can be substituted for valine (Val). The Met form of the COMT enzyme is less active and leads to higher levels of synaptic dopamine in the prefrontal cortex (Chen et al. 2004; Lotta et al. 1995). As the COMT gene is important for modulating dopamine levels, the *COMT* gene Val^158^Met functional polymorphism is associated with cognitive functioning and emotion regulation. Mier et al. (2010) have conducted a meta-analysis of fMRI studies that investigated the influence of *COMT* gene Val^158^Met polymorphism on prefrontal activation using cognitive processing tasks (working memory and memory processes) and emotional processing (affective stimuli) tasks. The authors of the meta-analysis (Mier et al. 2010) present opposing effects of the *COMT* gene Val^158^Met polymorphism on cognitive functioning and emotion processing.

In terms of cognitive processes, they (Mier et al. 2010) show that there is a significant association between the *COMT* genotype and the prefrontal activation and Met allele carriers show less activation (and enhanced processing). Studies showing Met allele carriers displaying better performance in various cognitive functions are numerous (Barnett et al. 2007, 2008; Bruder et al. 2005; De Frias et al. 2004; Starr et al. 2007). Similarly, the *COMT* gene has been related to several cognitive functions (Barnett et al. 2007, 2009; Diamond et al. 2004; Dumontheil et al. 2011), including autobiographical memory (Tõugu et al. 2016) in child samples. Preschool children who are Met/Met homozygotes provide more memory details when asked to report on experienced events than children who are Val/Met heterozygotes (Tõugu et al. 2016).

For emotional processing, the meta-analysis by Mier et al. (2010) revealed an association between the *COMT* genotype and prefrontal activation where carriers of Met alleles show stronger activation and reduced efficiency. Yet, the effect could be more complex. For example, on the one hand, Met alleles were correlated with more difficulties in verbalizing feelings (Swart et al. 2011). On the other hand, the Met allele has been related to faster emotion recognition (Tamm et al. 2016). Barzman et al. (2015) have proposed *COMT* gene Val^158^Met polymorphism as one of the possible genetic factors in emotion dysregulation in children and adolescents. And indeed, Val/Val homozygote adolescent girls have reported greater maintenance of positive emotions during stress (Waugh et al. 2009). In the same study, girls with a Val allele also reported greater perceived social acceptance.

At the same time, the genotype effect could be modified by gender or environmental factors. For example, infants with a Val allele who had mothers with a maltreatment history used a more maladaptive emotion regulation strategy in a stressful situation (Villani et al. 2018). Similarly, Val/Val genotype children with a history in institutional care score higher than their Met/Met or Val/Met genotype counterparts on depressive symptoms, especially when they remain in institutional care (as compared to being placed in foster homes) (Drury et al.^2010^). Also, carriers of the Val allele who perceive higher maternal rejection report higher levels of loneliness compared to Met/Met homozygotes (Wei et al. 2021). These studies show that Val/Val homozygotes could be particularly vulnerable to aversive environmental factors in childhood. In the cognitive domain, the effect of *COMT* gene Val^158^Met polymorphism has been shown to differ for the sexes. The gene polymorphism has a notable effect on boys’ cognitive performance, but no effect on girls’ performance (Barnett et al. 2007, 2009).

### The present study

The present study sets out to investigate the link between the quality and content of children’s early autobiographical memory and *COMT* gene Val^158^Met polymorphism. Autobiographical memory is a cognitive ability. Memory processes affect the formation of personal memories and childhood amnesia (Bauer 2015). At the same time, the quality of autobiographical memories is indicative of psychopathology as people diagnosed with depression often report scant and very general autobiographical memories (Sumner et al. 2010) and struggle with providing positive memories, in particular (Feurer et al. 2018). Understanding how the cognitive and emotional processes contribute to the formation of early memories would broaden our understanding of autobiographical memory and its mechanisms. Comprehension of these mechanisms could also have practical implications for boosting children’s resilience.

The *COMT* gene Val^158^Met polymorphism is related to cognitive functions and to early autobiographical memory development. This implies that *COMT* gene Val^158^Met polymorphism affects autobiographical memory formation via its influence on the cognitive processes. Children with a Met allele would have qualitatively better early memories to report.

In addition, since first autobiographical memories often include emotional content, the prospective effect of *COMT* gene Val^158^Met polymorphism on emotional processing could be apparent in children’s recollections. Since Met alleles are associated with reduced processing efficiency for emotional stimuli, remembering emotional events, in particular, could be enhanced in the group of children with a Val allele.

The present study aims to investigate the effect of *COMT* gene Val^158^Met polymorphism on the quality and emotional content of early memories of children. In order to do this, the quality of memory is defined by assessing the specificity of the reported event and calculating the number of reported details (both related to the event and additional evaluative information). The emotional content of the reported memory is explicated by assessing its valence and calculating the number of emotion words used in the report.

### Hypotheses

The *COMT* gene Val^158^Met polymorphism is expected to influence the valence of the remembered event, the specificity of the memory, the emotional content, and the number of details of children’s autobiographical memories. Based on the previous research, we formulate the hypotheses:

1. Met/Met homozygotes provide more specific and detailed first autobiographical memories overall than Val/Met heterozygotes and Val/Val homozygotes.
2. Met/Met homozygotes report events with a positive/negative valence less often and are less likely to use emotional words than children with Val/Met or Val/Val genotypes.
3. Girls recall (a) more emotional and specific events than boys and (b) provide more details and (c) more emotional content for their first autobiographical memories than boys.

Exploratory analyses are carried out regarding *COMT* gene Val^158^Met polymorphism interaction effects by gender and by valence on the event and regarding the effect of valence on the number of details reported.

## Method

### Participants

All children were participants in the large-scale longitudinal Estonian Children Personality, Behavior, and Health Study (Tomson et al. 2011). The present sample includes children who participated in the additional block devoted to the investigation of autobiographical memory. Data were collected in 2014 in Estonian schools. All participants were Caucasian and attendees of an Estonian-speaking school who had previously attended Estonian-speaking kindergartens. Overall, 321 schoolchildren aged 9–13 (*M* = 10.2, SD = .77) participated in the additional block: 160 of them were boys and 161 of them girls. Genotype information is available for 245 participating children (*M*_age_ = 10.2, SD = .77, 50.6% girls). Due to some missing data, the sample used for the analyses included 234 schoolchildren aged 9–13 (*M*_age_ = 10.2, SD = .77), 114 boys and 120 girls.

### Procedure and materials

Parents of the children in the longitudinal study were contacted to provide written consent. Children whose parents had provided consent were invited to partake in data collection at school during a lesson in a separate classroom. Participants were asked to fill in a written questionnaire individually. In the instruction of the autobiographical memory task, children were asked to recall the very first memory of their life that they could remember and write it down in as much detail as they could. They were also asked to indicate their age at the time of the remembered event, their age at present, and gender. A research assistant was present to answer questions if the child did not understand the task. The research assistant also made sure that the children did not talk to each other during the task. The task took about 20–25 min to complete.

### Genotyping

Blood samples for genotyping were collected from the participants already during the first wave of the study when they were 3–5 years old. Genomic DNA was extracted from dried blood spots using Qiagen QIAamp®DNA Mini Kit (Qiagen, Hilden, Germany). A standard procedure outlined by the manufacturer (Thermo Fisher Scientific 2012) was used for genotyping. Applied Biosystems™ TaqMan® SNP Genotyping Assays use TaqMan® 5´ nuclease chemistry for amplifying and detecting specific polymorphisms in purified genomic DNA samples. Each assay allows genotyping of individuals for a single-nucleotide polymorphism (SNP). All assays are developed using bioinformatics assay design processes that apply heuristic rules that are deduced from both manufacturing and assay performance data. Genotyping reactions of COMT Val^158^Met polymorphism (rs4680) were performed in a total volume of 20 μl with10–50 ng of template DNA as previously described (Lehto et al. 2013). The real-time polymerase chain reaction (RT-PCR) was performed with primers and fluorescent probes obtained from Applied Biosystems (Foster City, CA, USA) Custom TaqMan SNP Genotyping Assays. RT-PCR reaction components and final concentrations were as follows: 1:5 5 × HOT FIREPol Probe qPCR Mix Plus (ROX) (Solis BioDyne) and 1:20 80 × TaqMan Primers Probe (F 5′-CCCAGCGGATGGTGGAT-3′; R 5′-CAGGCATGCACACCTTGTC-3′; Reporter1-TTCGCTGGCATGAAG (VIC); Reporter 2-TCGCTGGCGTGAAG (FAM)). Reactions were performed on the ABI 7500 Real-Time PCR system, and the amplification procedure consisted of an initial denaturation step at 95 °C for 15 min and forty 15-s cycles at 95 °C and 1 min cycles at 60 °C. All genotyping reactions were carried out in duplicates, and extra negative controls were added to each reaction plate. No inconsistencies occurred. Genotypes were found to be in the Hardy–Weinberg equilibrium.

### Coding

In total, 321 children participated in the autobiographical memory task, but only the memories of children whose genotype information was available (*N* = 245) were analyzed further. Eleven of them either left the response sheet blank or wrote “I don’t remember.” Overall, 234 first memories of schoolchildren were analyzed. Two coders were trained for each of the coding schemes. The second coder was always the first author. The coder and the author coded 15% of the material in order to calculate intercoder reliability. All discrepancies related to coding the different aspects of the data were resolved in discussion, and the first author proceeded to code the rest of the data.

#### Memory quality: specificity and details

The memories that the children provided were categorized as specific or general, based on Nuttall et al. (2014). *Specific memories* (*N* = 183) described at least one particular event that did not last longer than a day; *general memories* (*N* = 28) included descriptions of routine events or prolonged periods, e.g., summer vacations. In addition, memories were coded as *semantic memories* (*N* = 23) if children described memories that they did not recall themselves, but what they had been told about, or where they listed life events without describing the event itself. Examples for all the categories are provided in Table 1. Intercoder agreement for coding the specificity of events was 94%.

**Table 1:** Examples of different types of memory narratives provided by the children

Type of memory	Examples of memory narratives provided
Specific memory	9-year-old girl: *When the Christmas tree caught on fire at Christmas because it was too dry for the sparkling candles. Everyone got really upset and was running around with their cups. It happened at home and we didn’t need the fire brigade*
	11-year-old boy: *My best friend gave me his guitar to play with in kindergarten. I played with it and I broke it by accident. I gave it back and said that another boy did it. The other boy got shouted at but I didn’t*
General memory	10-year-old boy: *I went to visit my aunt in Germany, but all I remember is my niece’s place and even there I only remember the ceiling*
	11-year-old boy: *I was home with my little brother. I played with him*
Semantic memory	10-year-old boy: *I was sleeping and my uncle put some dice on my head and a book in my hands*
	11-year-old boy: *I was lying on the floor and then my dog Patrick would give me his ears to help me get up. I don’t remember it myself, but my mum and dad tell me, he used to do it all the time*

Specific memories were further coded for episodic and extra-episodic details based on the coding scheme of Levine et al. (2002). *Episodic details* referred to the memory event described and included mentioning actions (“*I fell down the tree.*”), describing the circumstances of the event (“*It was dark.*”, “*The ship was blue and white.*”), and pointing out feelings, thoughts and evaluations (“*We were so scared.*”, “*It was nice.*”). *Extra-episodic details* were details that added semantic information (“*I was living in the country-side then.*”, “*Now the dog has died and we have a new one.*”) or provided information about another (often related) event. Intercoder agreement for coding details was 77%.

#### Emotional content

First, the reported memory events were categorized according to valence either as (1) *neutral* (memory events where no reference was made to the emotions experienced at the time and the event itself carried no positive or negative implications, e.g., playing with friends, going for an outing, etc.), (2) involving *negative emotion* (e.g., accidents, being scared or ill, etc.), or (3) involving *positive emotion* (e.g., birthday celebrations and events when the child indicated being happy or excited) (adapted from Peterson et al. 2005).

Second, all positive emotion words and negative emotion words were counted. Emotion words were words that expressed emotions or emotional actions or provided an emotional evaluation of the event (e.g., positive emotion words included words and expressions like “*I was excited*.” “*We laughed*.”, “*I loved it.*” etc., while examples of negative emotion words include “*I cried and yelled*.”, “*It was horrible*.”, “*We got scared*.” etc.). Intercoder agreement for emotional valence was 80%, and for emotion words 97%. Emotion words were very infrequent in children’s event memories (range 0–6, *M* = .75, SD = 1.02), and many children (*n* = 133) did not use any emotion words. In order to use the variable in the analyses, a dichotomous variable was created differentiating children who used at least one emotion word (*n* = 101) and children who did not use any emotion words (*n* = 133).

## Results

### Quality of first memories

The quality of first memories was assessed by specificity of the first memory and the number of details (episodic and extra-episodic) provided in the memory account. One hundred and eighty-three children provided a specific memory, and 51 children described a general memory or semantic memory as their first memory. On average, a specific memory provided by children included 7.52 (SD = 4.91) episodic details and 2.42 (SD = 3.54) extra-episodic details. Descriptive information about the first memories is provided in Tables 2 and 3.

**Table 2:** Descriptive statistics of children’s first memories for boys and girls with a different *COMT* Val^158^Met genotype

Gender	Genotype	% of children providing specific memories	% of children recalling negative or positive events	% of children using emotion words	Number of episodic details *M* (SD)	Number of extra-episodic details *M* (SD)
Boys	Val/Val (*n* = 30)	66.7	46.7	36.7	7.55 (4.92)	3.05 (3.36)
	Val/Met (*n* = 52)	75.0	51.9	26.9	5.23 (3.13)	1.15 (1.80)
	Met/Met (*n* = 32)	75.0	56.3	28.1	8.88 (4.53)	1.42 (1.91)
Girls	Val/Val (*n* = 19)	84.2	68.4	57.9	7.50 (3.54)	4.13 (3.83)
	Val/Met (*n* = 58)	82.8	72.4	56.9	8.69 (6.14)	3.52 (4.79)
	Met/Met (*n* = 43)	83.7	62.8	53.5	7.56 (4.81)	1.86 (3.20)

**Table 3:** Mean number of episodic and extra-episodic details provided for positive, negative, and neutral events

	Positive events *M* (SD)	Neutral events *M* (SD)	Negative events *M* (SD)
Number of episodic details	8.42 (5.60)	5.81 (3.54)	8.28 (5.02)
Number of extra-episodic details	3.42 (4.36)	1.61 (2.94)	2.30 (3.13)

In order to investigate the relationship between memory specificity, genotype, and gender (Hypotheses 1 and 3a), a three-way log-linear model including gender (Male/Female), *COMT* genotype (Met/Met, Val/Met, Val/Val), and memory type (Specific/Not Specific) was estimated. The final model retained the gender × memory-type interaction. The likelihood ratio of this model was *χ*^2^(6) = 5.06, *p* = .54, and the gender × memory-type interaction was significant (*χ*^2^ (1) = 4.73; *p* < .05). The odds ratio indicated that girls were 1.91 times more likely than boys to provide specific memories as their first memories.

**Fig. 1:**
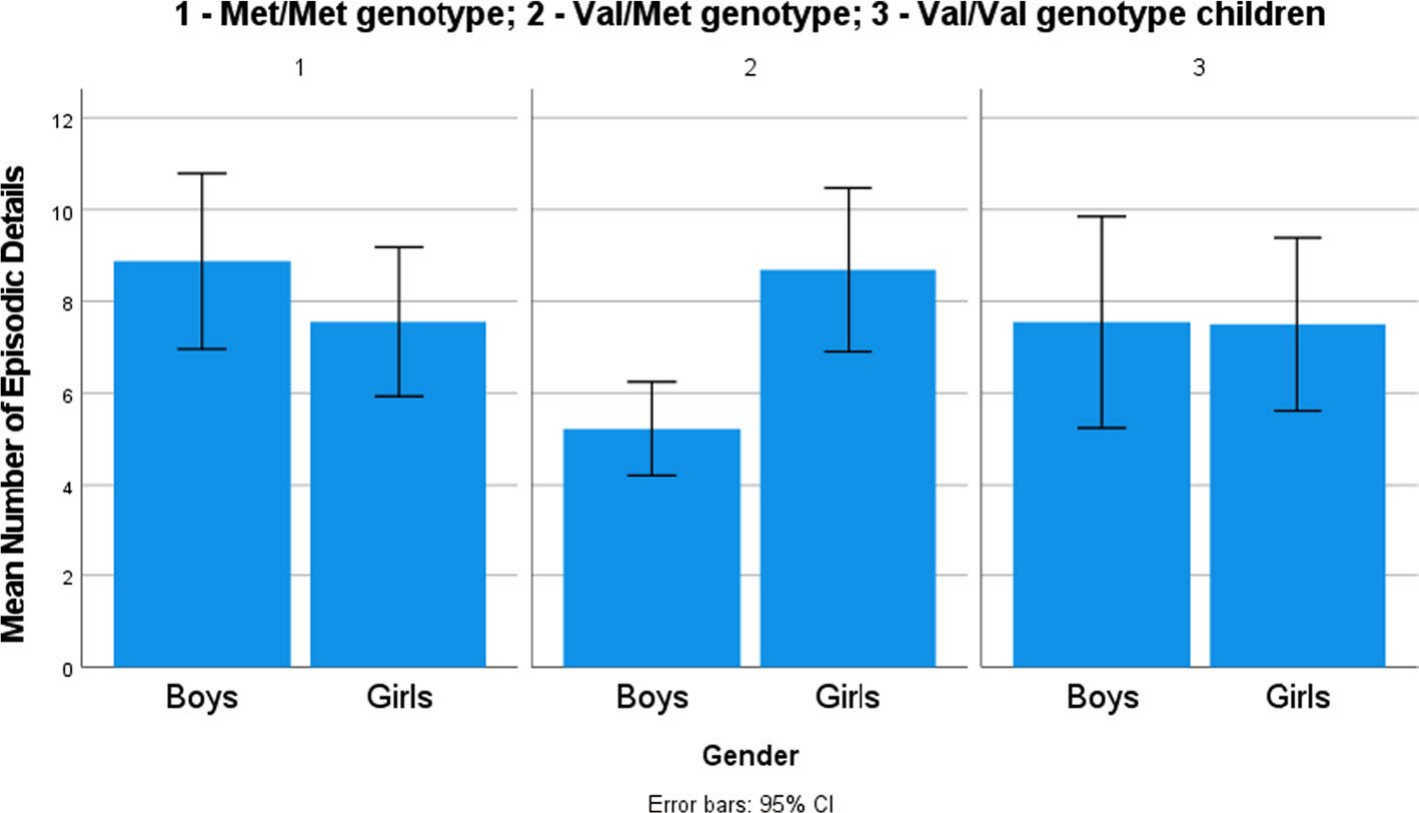
Mean number of episodic details provided by boys and girls with different genotypes

**Fig. 2:**
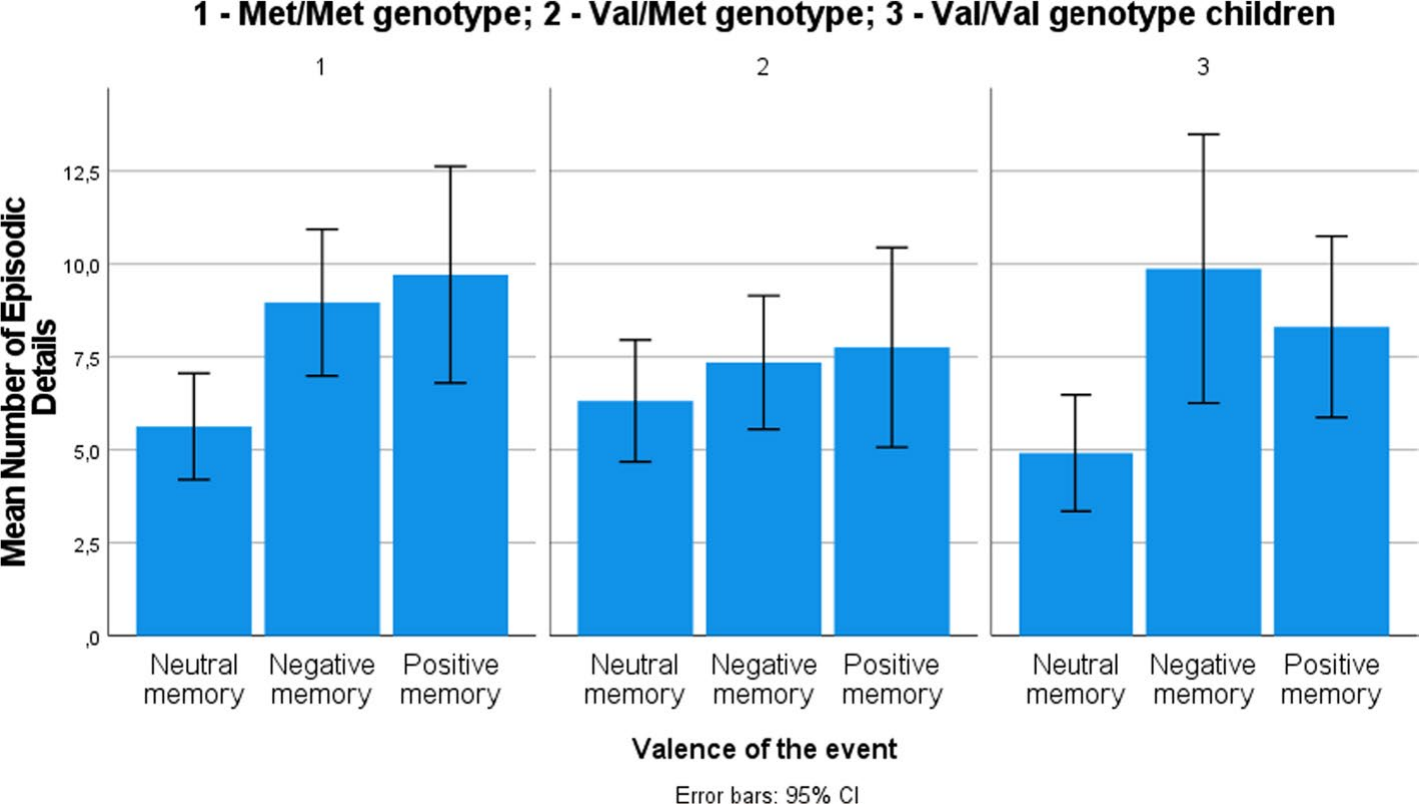
Mean number of episodic details provided by children with different genotypes recalling memories of different valence

In order to investigate the effect of genotype, gender (Hypotheses 1 and 3b), and the effect of the valence of the event (exploratory analysis) on the number of details reported by children, a generalized linear model for Poisson distribution with a log-linear link function was used. First, a model with the number of children’s episodic details as a dependent factor and gender, genotype, and valence of the event as independent factors was estimated. All two-way interactions were included in the model. The overall model was significant (goodness-of-fit deviance/df = 2.58; likelihood ratio *χ*^2^(13) = 90.31, *p* < .001), indicating that the independent variables were systematically related to the dependent variable. In the full model, gender categories did not differ in the contribution to the model, while Val/Met children provided more details than Val/Val children (*β* = .58, *χ*^2^(1) = 9.87, *p* < .01) and children provided more details for both positive (*β* = .60, *χ*^2^(1) = 10.13, *p* = .001) and negative events (*β* = .81, *χ*^2^(1) = 6.43, *p* = .01) compared to neutral events (see descriptive details in Table 3). Regarding interactions, Val/Met genotype boys provided fewer details (*β* = − .62, *χ*^2^(1) = 15.78, *p* < .001) than other children (see Fig. 1) and Val/Met children reported fewer details for positive (*β* = − .45, *χ*^2^(1) = 5.12, *p* < .05) and negative events (*β* = − .61, *χ*^2^(1) = 8.75, *p* < .01) than other children (see Fig. 2). Main effects and interactions are provided in Table 4.

**Table 4:** Results for the generalized linear models for Poisson distribution with a log-linear link function for the number of episodic details and extra-episodic details provided by children

Main effects and interactions	Model for episodic details			Model for extra-episodic details		
	*Wald χ*^*2*^	*df*	*p*	*Wald χ*^*2*^	*df*	*p*
Intercept	4109.45	1	.00	182.71	1	.00
Gender	.98	1	.32	**16.67**	**1**	**< .001**
COMT	**7.99**	**2**	**.02**	**24.96**	**2**	**< .001**
Valence of the event	**37.51**	**2**	**< .001**	**14.52**	**2**	**< .001**
Gender × COMT	**32.95**	**2**	**< .001**	**13.08**	**2**	**.001**
Gender × valence of the event	.82	2	.66	1.87	2	.39
COMT × valence of the event	**13.94**	**4**	**.01**	**29.27**	**4**	**< .001**

All significant results (*p* < .05) are indicated in bold

**Fig. 3:**
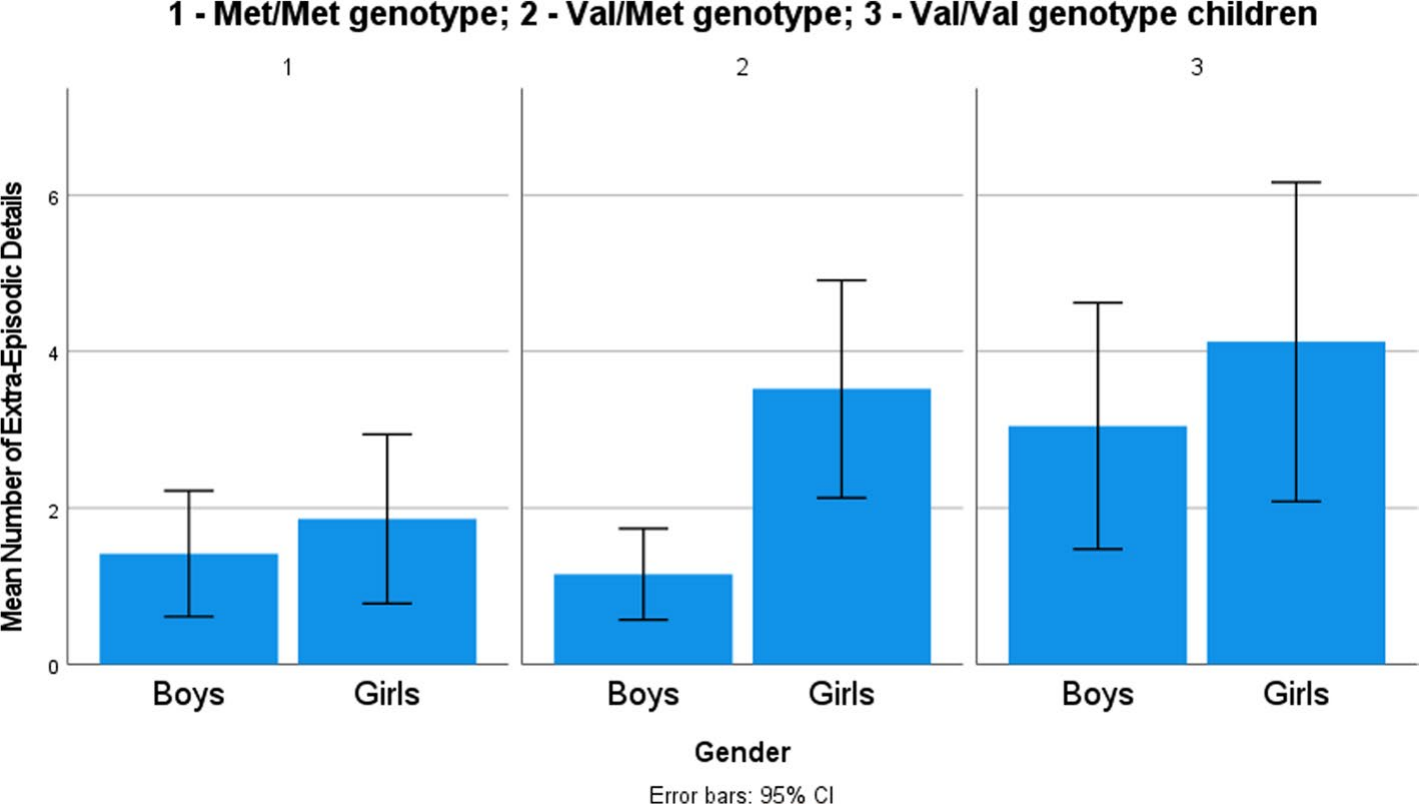
Mean number of extra-episodic details provided by boys and girls with different genotypes

**Fig. 4:**
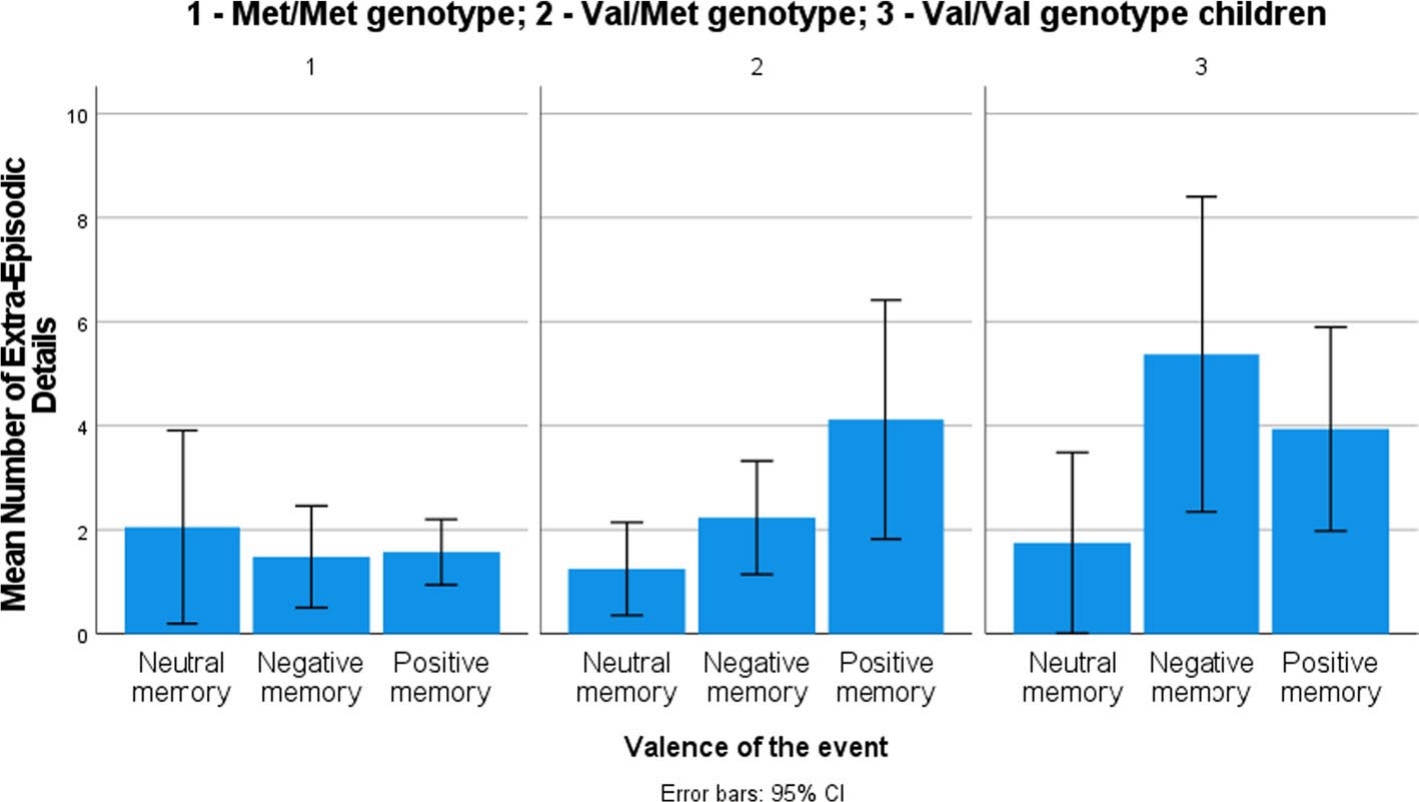
Mean number of extra-episodic details provided by children with different genotypes recalling memories of different valence

Second, a similar model was estimated with extra-episodic details as a dependent variable and gender, genotype (Hypotheses 1 and 3b), and valence of the event (exploratory analysis) as independent factors. All two-way interactions were included in the model. The overall model was significant (goodness-of-fit deviance/df = 3.63; likelihood ratio *χ*^2^(13) = 143.24, *p* < .001), indicating that the independent variables were systematically related to the dependent variable. In the full model, gender categories and COMT categories did not differ in the contribution to the model; children provided more extra-episodic details for both positive (*β* = .79, *χ*^2^(1) = 6.43, *p* = .01) and negative events (*β* = 1.20, *χ*^2^(1) = 14.43, *p* < .001) compared to neutral events (see descriptive information in Table 3). Regarding interactions, Val/Met genotype boys provided fewer extra-episodic details (*β* = − .82, *χ*^2^(1) = 10.56, *p* = .001) than Val/Met genotype girls (see Fig. 3) and Met/Met genotype children reported fewer extra-episodic details for positive (*β* = − 1.08, *χ*^2^(1) = 7.95, *p* < .01) and negative events (*β* = -1.38, *χ*^2^(1) = 14.45, *p* < .001) as compared to other children and neutral events (see Fig. 4). The main effects and interactions are provided in Table 4.

### Emotional content of first memories

The emotional content was assessed by the general valence of the reported memory and the use of emotion words in the memory account. Neutral events were reported as their first autobiographical memories by 93 children, 76 children recalled negative events, and 65 positive events. At least one emotion word was used in the memory account by 101 children, while 133 children did not use any emotion words. In order to investigate the relationship between emotional valence, genotype, and gender (Hypotheses 2 and 3a), a three-way log-linear model including gender (Male/Female), *COMT* genotype (Met/Met, Val/Met, Val/Val), and memory valence (Negative/Neutral/Positive) was built. The analysis produced a final model that retained the gender × memory valence interaction. The likelihood ratio of this model was *χ*^2^(6) = 12.68, *p* = .24, and the gender × memory valence interaction was significant (*χ*^2^ (2) = 8.18; *p* < .05). The cross-tabulation showed that 31.7% of girls provide a neutral memory compared to 48.2% of boys doing so and 35% of girls provide a positive memory compared to 20.2% of boys doing so.

In order to investigate the effect of genotype and gender on the use of emotion words by children (Hypotheses 2 and 3c), a three-way log-linear model including gender (Male/Female), COMT genotype (Met/Met, Val/Met, Val/Val), and the use of emotion words (Yes/No) was built. The analysis produced a final model that retained the gender × use of emotion words interaction. The likelihood ratio of this model was *χ*^2^(6) = 5.34, *p* = .50, and the gender × use of emotion words interaction was significant (*χ*^2^ (2) = 16.35; *p* < .001). The odds ratio indicated that boys were 2.9 times less likely than girls to include emotion words in their memory accounts.

## Discussion

The present study set out to establish *COMT* gene Val^158^Met polymorphism effects on the quality and emotional content of Estonian schoolchildren’s first autobiographical memories. In addition, gender effects were considered and the emotional valence of the first memory was taken into account.

### *COMT* gene effects

The expected effects of *COMT* gene Val^158^Met polymorphism regarding the specificity and emotional content (Hypotheses 1 and 2) were not confirmed in the study. Children with a different genotype were equally likely to recall a specific memory as compared to a general memory or a positive or negative memory as compared to a neutral memory, and to use emotion words in their memory reports. One of the reasons that the main effects of the genotype were not revealed could be children’s age. Prior research has indicated that the effects of *COMT* gene Val^158^Met polymorphism on cognitive functions are stronger after puberty (Barnett et al. 2007; Gaysina et al. 2013). Therefore, a study with older children could still reveal main effects of genotype on first autobiographical memories. At the same time, other studies have indicated that the *COMT* genotype effect may be dependent on the environment (Drury et al. 2010, Tõugu et al. 2016) or gender of the child (Barnett et al. 2007, 2009). In the present study, *COMT* genotype interaction with gender and valence of the event appeared (see discussion below).

### Gender effects

The hypothesis regarding gender effects (Hypotheses 3a and c) was to a large extent supported by the data. We found that girls were almost two times more likely than boys to provide specific memories. In addition, girls provided more emotional memories than boys did: They were more likely than boys to report positive events and include emotion words in their memory narratives.

The gender effects revealed in the present study largely corroborate prior findings. Earlier findings show that women are more likely to provide specific memories than men (Pillemer et al. 2003) and that the first memories of women and girls are more emotional than the first memories of boys and men (Buckner and Fivush 1998; Mullen 1994; Rice and Pasupathi 2010). This gender effect could be due to gender-specific autobiographical memory socialization that takes place during the early years (Buckner and Fivush 1998). Socialization seems to have a significant effect on autobiographical memory development as certain socialization patterns lead to earlier and more detailed first memories (Jack et al. 2009). The gender-specific socialization is apparent, for example, when parents’ reminiscing conversations with daughters are more detailed (Reese and Fivush 1993; Reese et al. 1996) and focus more on the emotional content than their conversations with sons (Adams, et al. 1995; Fivush et al. 2000). At the same time, it cannot be ruled out that girls may also notice and/or report emotional aspects of events more readily.

At the same time, the hypothesis about gender differences in the number of details (both episodic and extra-episodic) provided by children (Hypothesis 3b) was not supported. Gender seemed to have a significant main effect in the model, but when all the main effects and interactions were considered together, boys and girls did not differ in the number of details provided. Instead, there were interactions between gender and *COMT* gene Val^158^Met polymorphism regarding the number of details of first memories (see discussion below).

### Memory details and valence of the event

Regarding exploratory investigations, the quality of first memories in terms of details provided was related to the valence of the experienced event. Children provided more episodic and extra-episodic details when recounting emotional (positive and negative) events as compared to neutral events. An earlier study by Pasupathi and Wainryb (2010) has found that negative memories of children are more detailed than positive ones. That particular study did not include neutral events, but probed for positive and negative memories. Therefore, it is possible that in free recall children’s memories of negative and positive events are similarly detailed as compared to neutral recollections that contain fewer details.

The episodic and extra-episodic details of a memory serve different functions: Episodic details relate to the quality of the memory, while extra-episodic details provide evaluation of the event and personal importance to the teller (Bauer and Larkina 2014) and serve to interpret the memory (Pasupathi and Wainryb 2010). Based on the current findings, it is not clear if children, in fact, remembered more details because the event was emotional or emotional events supply more details to tell (i.e., talk about the emotions and other’s reaction to it, etc.). However, since both episodic and extra-episodic details are more plentiful for emotional memories, it could be both. In the present study, talk about emotions and emotional actions in the memory accounts was rare. This finding seems to render some support to the idea that emotional events are more memorable as children provide more details about them in general. Yet, while children also provide more extra-episodic details about emotional memories, it could be that these memories are more significant to them and make better stories to tell, as there is additional (evaluative and interpretive) information to add.

### *COMT* gene interactions with valence of the event and gender

The *COMT* gene interaction with gender and valence of the event were significant regarding the number of details (both episodic and extra-episodic) provided by the children. Most of the interactions uncovered involved heterozygote children: Val/Met genotype boys provided fewer details (both episodic and extra-episodic) than girls with the same genotype did, and Val/Met children reported fewer details for positive and negative events compared to other children and neutral events. In addition, Met/Met homozygote children provided fewer extra-episodic details regarding emotional events than other children did.

An earlier study with the same children (when the children were 4 and 6 years old) revealed that both girls and boys with a Val/Met genotype provided fewer details in general when reporting on past events (Tõugu et al. 2016). Now, at the mean age of ten, the effect persisted for emotional memories as compared to neutral ones and for boys as compared to girls with the same genotype. Earlier studies have found that the effect of *COMT* gene Val^158^Met polymorphism on cognitive functions is sometimes only observable in the sample of boys (Barnett et al. 2007, 2009). Hence, the genotype by gender interaction could reflect a trajectory of general autobiographical memory development. At the same time, there could be some general cognitive mechanism involved affecting the Val/Met heterozygote group as several birth cohort representative studies have found that COMT heterozygotes have a large gender gap in education (Kurrikoff et al. 2018; Lehto et al. 2013). In both studies, Val/Met heterozygote men have significantly lower educational attainments than Val/Met heterozygote women in Estonia (Kurrikoff et al. 2018; Lehto et al. 2013).

Alternately, Val/Met genotype carriers could be more vulnerable to environmental factors than other genotypes. An earlier study (Tõugu et al. 2016) reported that Val/Met heterozygote children of mothers who did not have a university degree provided fewer details than Met/Met homozygote children of mothers with a similar educational attainment (at the age of 4, and not later). It is possible that Val/Met heterozygote children aged 9–13 are particularly sensitive to the gender typical socialization discussed earlier.

Interestingly, Met/Met homozygote children provided the smallest amount of extra-episodic or additional interpretive information regarding positive and negative events. As the extra-episodic information is often evaluative and marks the significance of the event for the teller, this result could suggest the differences in emotional processing the study set out to find. Mier et al. (2010) have linked the Met allele with reduced efficiency in emotional processing, and Waugh et al. (2009) have reported positive effects of Val/Val genotype on emotional processing in stressful events for adolescent girls. In the present study, Val/Met heterozygote and Val/Val homozygote children provided more extra-episodic information regarding emotional events than Met/Met homozygote children did; the difference is not detected for neutral events. This could indicate that for emotional events, the evaluative and self-significant information is more readily available to children with a Val allele.

The reasons for *COMT* gene interactions with valence of the event and gender remain to be learnt. Further studies of memory details with environmental variables taken into account are needed in order to unravel the reasons for gender- and valence-related differences. It would be very informative to see what the *COMT* gene Val^158^Met polymorphism effect on autobiographical memory would be in the adult sample.

## Conclusions

The present study investigated the effects of *COMT* gene Val^158^Met polymorphism and gender on the valence, specificity, details, and emotion words of Estonian schoolchildren’s first autobiographical memories. Overall, it appears that the *COMT* gene Val^158^Met polymorphism effect on the cognitive and emotional processing involved in autobiographical memory formation is not as clear-cut as implied in laboratory-based studies (see Mier et al. 2010). Regarding the quality and emotional content of autobiographical memories, there are *COMT* gene Val^158^Met polymorphism interactions by gender and by the valence of the remembered event. In ecologically valid situations, certain genotypes seem to render the carrier more susceptible to environmental factors (see also Drury et al. 2010; Wei et al. 2021). In the case of autobiographical memory, socialization or gender alongside genetic factors could influence the readiness to notice, incorporate, or report details related to emotional content of events and self-significance of the memory. This provides room for practical implications and opportunities for interventions that could possibly counterbalance unfortunate circumstances. Therefore, more studies are needed in order to determine the mechanisms of autobiographical memory development, to understand how the genotype polymorphism affects further development of autobiographical memory, and to clarify how it relates to the individual differences in autobiographical memory established in adulthood.
